# BfpI, BfpJ, and BfpK Minor Pilins Are Important for the Function and Biogenesis of Bundle-Forming Pili Expressed by Enteropathogenic Escherichia coli

**DOI:** 10.1128/JB.00818-15

**Published:** 2016-02-12

**Authors:** Claudia F. Martinez de la Peña, Leon De Masi, Shahista Nisa, George Mulvey, Jesse Tong, Michael S. Donnenberg, Glen D. Armstrong

**Affiliations:** a Department of Microbiology, Immunology and Infectious Disease, University of Calgary, Calgary, Alberta, Canada; b Division of Infectious Diseases, Department of Medicine, University of Maryland, School of Medicine, Baltimore, Maryland, USA; Princeton University

## Abstract

Enteropathogenic Escherichia coli (EPEC) remains a significant cause of infant diarrheal illness and associated morbidity and mortality in developing countries. EPEC strains are characterized by their ability to colonize the small intestines of their hosts by a multistep program involving initial loose attachment to intestinal epithelial cells followed by an intimate adhesion phase. The initial loose interaction of typical EPEC with host intestinal cells is mediated by bundle-forming pili (BFP). BFP are type 4b pili (T4bP) based on structural and functional properties shared with T4bP expressed by other bacteria. The major structural subunit of BFP is called bundlin, a T4b pilin expressed from the *bfpA* gene in the BFP operon, which contains three additional genes that encode the pilin-like proteins BfpI, BfpJ, and BfpK. In this study, we show that, in the absence of the BFP retraction ATPase (BfpF), BfpI, BfpJ, and BfpK are dispensable for BFP biogenesis. We also demonstrate that these three minor pilins are incorporated along with bundlin into the BFP filament and contribute to its structural integrity and host cell adhesive properties. The results confirm that previous findings in T4aP systems can be extended to a model T4bP such as BFP.

**IMPORTANCE** Bundle-forming pili contribute to the host colonization strategy of enteropathogenic Escherichia coli. The studies described here investigate the role for three minor pilin subunits in the structure and function of BFP in EPEC. The studies also suggest that these subunits could be antigens for vaccine development.

## INTRODUCTION

The main virulence feature of the enteropathogenic Escherichia coli (EPEC) group of enterovirulent E. coli is their ability to produce attaching-and-effacing (A/E) lesions on the epithelial lining of the human small intestine (1). These lesions result from the rearrangement of the actin cytoskeleton, leading to the loss of microvilli and, subsequently, intimate attachment of the organisms to host intestinal epithelial cells (2). Typical EPEC strains differ from the atypical strains by their expression of bundle-forming pili (BFP), which are classified as type 4 pili (T4P). T4P are thin (50 to 80 Å in diameter), flexible filaments that have been classified into the groups T4aP and T4bP depending on the structure of their major pilin subunits. The BPF expressed by EPEC are members of the T4bP group, and the main pilin subunit is called bundlin (3). BFP assist the A/E process by promoting the formation of microcolonies and the early, nonintimate attachment of the organisms to host cells. This process is referred to as localized adherence (LA) (4, 5).

One functional feature of the T4aP group is their ability to extend from, and retract back into, the cell, a process energized by ATP hydrolysis (6, 7). In addition to participating in the early nonintimate phase of EPEC adherence to host intestinal cells, T4aP have been implicated in such processes as immune evasion, twitching motility, biofilm development, autoaggregation, and phage attachment (8–14). T4aP and T4bP pilins are synthesized as prepilin proteins, which are processed by a specific prepilin peptidase in the cytoplasmic membrane. All T4aP and T4bP pilins have a hydrophobic N-terminal segment (6 to 7 and 10 to 30 residues in length, respectively), and many also possess a methylated N-terminal amino acid residue (15). Despite the sequence variation found in the C-terminal regions of T4aP and T4bP pilins, many possess a pair of conserved cysteine residues (16) that are oxidized to form a loop structure which contributes to pilus stability and function (17).

In EPEC, the BFP-encoding genes reside in an operon on the EPEC adherence factor (EAF) plasmid. In addition to *bfpA*, the gene that encodes bundlin (18), there are three additional genes (*bfpI*, *bfpJ*, and *bfpK*) which encode pilin-like proteins that share many structural features with bundlin, including the prepilin peptidase cleavage site (19), the hydrophobic N-terminal sequence, and the conserved cysteines in their C-terminal domains. The ATPase enzymes responsible for the BFP extension and retraction processes are encoded by *bfpD* and *bfpF*, respectively. Moreover, although *bfpD* is absolutely required for BFP expression, the *bfpF* gene is not, and retraction-defective *bfpF* deletion mutants display a hyperadherent phenotype relative to that of the wild-type (WT) parental EPEC strain (20).

The role of BfpI, BfpJ, and BfpK in BFP structure and function still is poorly defined, because single-deletion mutants lacking any of the *bfpI*, *bfpJ*, and *bfpK* genes do not express BFP (19). As a result, these mutants do not exhibit a phenotype that can be attributed to any one of the pilin-like subunits (19). However, in the present study we determined that EPEC pilin-like deletion mutants, like those produced in Pseudomonas aeruginosa (21), Neisseria gonorrhoeae (22), and Neisseria meningitidis (23), do express BFP if they are created in a *bfpF* retraction-ATPase-deficient background strain. This study used this finding to describe the role of BfpI, BfpJ, and BfpK in BFP structure and function, and the results are presented here.

## MATERIALS AND METHODS

### Bacterial strains, plasmids, and growth conditions.

The bacterial strains, plasmids, and PCR primers used in these investigations are described in Tables 1 and 2. Bacteria were routinely grown overnight in Luria broth (LB) or tryptic soy broth (TSB) without dextrose at 37°C from single colonies picked from an overnight LB agar plate. Ampicillin (100 μg/ml), kanamycin (Km; 50 μg/ml), tetracycline (12.5 μg/ml), or chloramphenicol (25 μg/ml) was added to the media when required for mutant selection purposes. The complemented plasmids for EPEC mutants were induced using 1 mM isopropyl-β-d-thiogalactopyranoside (IPTG). Bacteria also were grown in Dulbecco's modified Eagle's medium (DMEM) (Invitrogen) supplemented with 44 mM NaHCO_3_ and 25 mM glucose at 37°C or on tryptic soy agar (TSA) agar plates supplemented with 5% (vol/vol) defibrinated sheep blood (blood agar plates [BAP]) to induce BFP expression (3). All DNA procedures were performed using standard genetic and molecular techniques (29). Plasmid DNA and RNA were purified using Qiagen kits (Qiagen Inc.). Restriction and other DNA-modifying enzymes were obtained from New England BioLabs Inc. and were used according to the supplier's instructions.

**TABLE 1:** Strains and plasmids used in this study

Strain or plasmid	Description, genotype, or sequence	Reference or source
Strains		
E2348/69	Wild-type EPEC strain, serotype O127:H6	24
UMD946	E2348/69 Δ*bfpF*	25
UMD951	E2348/69 Δ*bfpI*	This study
UMD957	E2348/69 Δ*bfpK*	This study
CMP-J	E2348/69 Δ*bfpJ*	This study
UMD963	E2348/69 *bfpF* Δ*bfpI*	This study
JPN15	E2348/69 cured of EAF plasmid	26
BL21(DE3) pLysS	F^−^ *dcm ompT hsdS*(r_B_^−^ m_B_^−^)	Novagen
CMP-1	E2348/69 Δ*bfpF* Δ*bfpI*	This study
CMP-2	E2348/69 Δ*bfpF* Δ*bfpJ*	This study
CMP-3	E2348/69 Δ*bfpF* Δ*bfpK*	This study
CMP-4	E2348/69 Δ*bfpIJK*	This study
CMP-5	E2348/69 Δ*bfpF* Δ*bfpIJK*	This study
Plasmids		
pET48b+	T7 promoter, His tag, N-terminus Trx tag	Novagen
pQE31	Vector for expressing N-terminally 6× His-tagged proteins	Qiagen
pMPM-T3	Low-copy-number cloning vector; p15A derivative; Tc^r^	27
pKD46	λ Red recombinase system plasmid	28
pKD4	Km cassette template plasmid	28
pETBfpI	*bfpI* cloned in pET48b+	This study
pETBfpJ	*bfpJ* cloned in pET48b+	This study
pQEBfpK	*bfpK* cloned in pQE31	This study
pT3-BfpI	pMPM-T3 derivative carrying *bfpI*	This study
pT3-BfpJ	pMPM-T3 derivative carrying *bfpJ*	This study
pT3-BfpK	pMPM-T3 derivative carrying *bfpK*	This study

**TABLE 2:** Primers used in this study

Primer	Sequence
*bfpI* F cloning in pET48b+	5′ ATTGGATCCGGTTATTGTGCTTGTTCAG 3′
*bfpI* R cloning in pET48b+	5′ ATTGAATTCCGCTTTCTTTTCTTATCA 3′
*bfpJ*FBamHI cloning in pET48b+	5′ TCTCTTTCTGTGGATCCCTATTACAAC 3′
*bfpJEcoR*I cloning in pET48b+	5′ TCTCTTTCTGTGGATCCCTATTACAAC 3′
F*bfpK* BamHI cloning in pQE31	5′ TTGTCTGTGGATCCTATTTCTGGCG 3′
*bfpK*RHIII cloning in pQE31	5′ ACACCAGAAGAAAAGCTTAACTAGTACCGT 3′
*bfpJ*pKD4F λ red recombination	5′ TCTTTTCTTTTACAATAATTTCCGGGTGGATGTTTTATGATAAGAGTGTAG GCTGGAGCTGCTTC 3′
*bfpJ*pKD4R λ red recombination	5′ ATCGCTATTTCAATAAGCGACAGTCCCTTTTGCTGACGTCCTTCAAAAAT CATATGAATATCCTCCTTAG 3′
*bfpK*pKD4 λ red recombination	5′ ATTCAGACATCAGGGCAAAATTGTGACAGAGGTGGATATGAT TTTTGAAGGACGTCAGGTGTAGGCTGGAGCTGCTTC 3′
*bfpK*pKD4R λ red recombination	5′ AAAATGGATACAATTAATGAAAAAAAACCACACCAGAAGAATAA CATATGAATATCCTCCTTAG 3′
*bfpI*pK λ red recombination	5′ AATATGTAAGTAAAAATTATGGTTCGTCTATGTTTATATTG AGAGTGTAGGCTGGAGCTGCTTC 3′
*bfpIFcl* qPCR	5′ TTAAGTATGCTGTTCATGAAAATGC 3′
*bfpIRcl* qPCR	5′ ACATCCACCCGGAAATTATTGTAAA 3′
*bfpJF* qPCR	5′ GGAGCGCAGAACAACATTATATG 3′
*bfpJR* qPCR	5′ CACAATTTTGCCCTGATGTCTGAA 3′
*bfpKinF* qPCR	5′ TTGTAGCAACAGATATGAATTC 3′
*bfpKinR* qPCR	5′ CACTTAATAACTCTAACGGGAG 3′
*rpoAF* qPCR	5′ GGCTTGACGATTTCGACATC 3′
*rpoAR* qPCR	5′ GGTGAGAGTTCAGGGCAAAG 3′
*bfpAF* qPCR	5′ TGATTGAATCTGCAATGGTG 3′
*bfpAR* qPCR	5′ AGCATTCTGCGACTTATTGG 3′
*BfpIHinDIIIF* clone in pMPMT3	5′ TACAGCCAAGCTTTACATCAAAGGAATATGT 3′
*bfpIBamHIR* clone in pMPMT3	5′ AAGGATCCTTTCTTTTCTTATCATAAAACA 3′
*bfpJSmaIF* clone in pMPMT3	5′ CTTTTACAATAATTCCCGGGTGG 3′
*bfpJSacIR* clone in pMPMT3	5′ GTCCCTTTTGCTGAGCTCCTTCAAA 3′
*bfpKSmaIF* clone in pMPMT3	5′ TTCAGACACCCGGGCAAAATTGTGA 3′
*bfpKXbaIR* clone in pMPMT3	5′ CCACTCTAGAAGAATAACATAACTA 3′

### Construction of EPEC mutants and complemented strains.

The EPEC mutants UMD963, UMD951, UMD957, CMP-J, CMP-1, CMP-2, CMP-3, CMP-4, and CMP-5 were constructed using the lambda red recombinase protocol described by Datsenko and Wanner (28). Briefly, the substitution of the gene of interest, *bfpI* (deleted from position 15 to 527), *bfpJ* (deleted from position 9 to 529), or *bfpK* (deleted from position 21 to 488), was accomplished using the pKD4 vector as a template by PCR amplifying the *aph* gene, encoding kanamycin resistance, using primers complementary to part of the Km cassette sequence and to part of the upstream and downstream regions of the target genes. The resulting PCR products containing the *aph* gene with flanking regions of the target genes then were transformed into either the EPEC wild-type strain E2348/69 or the EPEC *bfpF* mutant strain (UMD946) containing pKD46, encoding the lambda red proteins under the control of an arabinose-inducible promoter. The transformed colonies were selected by growing them at 37°C on LB plates supplemented with Km, and the desired mutations were verified by PCR and sequencing. The *bfpI*-, *bfpJ*-, and *bfpK*-complemented strains were created by cloning the amplified regions of each of these genes into pMPM-T3 using BamHI/HindIII for *bfpI*, SmaI/SacI for *bfpJ*, and SmaI/XbaI for *bfpK* restriction sites.

### RNA extraction and quantitative reverse transcription-PCR (RT-PCR).

Total DNA-free RNA was prepared from the wild-type EPEC strain E2348/69 and EPEC mutant strains grown in DMEM to an optical density at 600 nm (OD_600_) of 0.5. RNA subsequently was extracted using the Qiagen RNA extraction kit combined with DNase treatment to remove residual DNA from the preparations. One microgram of RNA was used to synthesize cDNA using qScript cDNA SuperMix (Quanta). The cDNA next was diluted 10-fold and used to perform real-time quantitative PCR with PerfeCTa SYBR green FastMix for iQ (Quanta) and specific primers for the *bfpA*, *bfpI*, *bfpJ*, and *bfpK* genes. The *rpoA* gene was used as a calibration standard to normalize the resulting data. The relative expression values, determined by *R* = 2^−(Δ*CT*)^ (where *C_T_* is threshold cycle), were calculated using the formula Δ*C_T_* = *C_T_* (*bfp* gene) − *C_T_* (*rpoA* gene).

### eLA assay.

The early localized adherence (eLA) assay was performed as described previously (30, 31), with slight modifications. Briefly, subconfluent HEp-2 cell monolayers were grown for 24 h on 8-well slides (Lab-Tek chamber glass slide system) at 37°C in a humidified atmosphere of 5% CO_2_ in minimal essential medium (MEM) supplemented with 10% fetal bovine serum (FBS). Prior to each experiment, 40 μl of TSB (without dextrose)-grown bacteria was inoculated into 4 ml of DMEM supplemented with 0.5% glucose in a CO_2_ incubator for 60 min to induce BFP expression. These cultures then were applied onto subconfluent HEp-2 cell monolayers at a multiplicity of 100:1 (bacteria to HEp-2 cells), and the slides were placed in the 37°C CO_2_ incubator for 45 min. The wells were washed five times with Na^+^/K^+^ phosphate-buffered (pH 7.2) physiological saline (PBS) to remove unbound bacteria, fixed with methanol for 10 min, and stained with Giemsa stain for 20 min. EPEC eLA was recorded using a phase contrast light microscope. A total of 10 fields and approximately 150 to 200 individual HEp-2 cells were examined, and those having attached microcolonies consisting of five or more bacteria were recorded as positive for eLA EPEC. The results are expressed as a percentage of cells positive for eLA relative to the total number of cells examined. Each experiment was performed in triplicate and repeated at least three independent times.

### Protein purification and antibody production.

Purified BfpI, BfpJ, and BfpK were obtained by amplifying and cloning their genes from EPEC strain E2348/69 lacking the DNA sequence encoding the first 25 amino acids from the N-terminal portion to enhance solubility. These then were cloned into the pET48b^+^ vector (Novagen) using the BamHI and EcoRI restriction sites for *bfpI* and *bfpJ* and into the pQE31 vector (Qiagen) for *bfpK* using the BamHI/HindIII restriction site. The pET48b+ plasmids then were transformed into E. coli BL21 cells, and protein expression was induced using 1 mM IPTG. The pQE31 vector was transformed into E. coli M15 cells, and its expression was induced using 1 mM IPTG. The three proteins were purified using nickel-nitrilotriacetic acid (Ni-NTA)–agarose (Qiagen) by following the manufacturer's protocol. New Zealand White rabbits were immunized using 100 μg of purified BfpI, BfpJ, or BfpK emulsified in 0.5 ml complete Freund's adjuvant by following the University of Calgary Health Sciences Animal Welfare Committee approved protocol M09030. The animals were given 2 booster immunizations at 3- and 4-week intervals using 50 μg purified proteins emulsified in incomplete Freund's adjuvant. After immunization, the animals were euthanized by following the standard procedure recommended by the Canadian Council on Animal Care (1993) and exsanguinated by cardiac puncture. To remove interfering antibodies and reduce background labeling, the resulting antisera were extensively absorbed against E. coli BL21.

### TEM and immunogold labeling.

Bacteria were grown overnight at 37°C on BAP to induce BFP expression. Single colonies then were carefully picked from the agar and gently suspended in a solution consisting of 2% formaldehyde plus 0.5% glutaraldehyde in 50 mM cacodylate buffer (pH 7.4). The fixed samples subsequently were deposited onto Formvar/carbon-coated copper electron microscope grids and stained with 1% phosphotungstic acid in double-distilled/deionized water. Images were obtained using a Hitachi H-7650 transmission electron microscope (TEM).

For immunogold labeling, bacteria were grown overnight at 37°C on BAP. Several EPEC colonies then were carefully picked from the plates and gently suspended in 100 μl of chilled (4°C) fixative solution. Twenty microliters of each preparation next was applied to Formvar/carbon-coated nickel grids (Electron Microscopy Science), which were allowed to remain undisturbed for 15 min before subsequent processing. Excess liquid was gently removed and the grids were incubated for 20 min with 20 μl of 50 mM glycine dissolved in PBS. The glycine-PBS solution was removed and 20 μl of 3% skim milk plus 0.01% Tween 20 in PBS was applied. The grids were allowed to remain undisturbed for 30 min. After 3 washes, with 5 min between each wash and 20 μl of incubation buffer (0.01% BSA-c [Aurion] in PBS), the grids were incubated for 60 min with the rabbit polyclonal anti-BfpI, anti-BfpJ, anti-BfpK, or anti-purified BFP (a kind gift from Jorge Girón, Centro de Deteccion Biomolecular, VIEP-BUAP, Puebla, Mexico) or preimmune sera diluted 1:100 in incubation buffer. The grids then were washed 6 times with incubation buffer. A goat anti-rabbit IgG gold-conjugated (10- or 15-nm particle size; Electron Microscopy Science) secondary antibody was diluted 1:25 in incubation buffer and applied to the grids for 45 min. The grids then were washed 6 times with incubation buffer and an additional 3 times with double-distilled/deionized water. Finally, the grids were stained for 10 s with 1% phosphotungstic acid in double-distilled/deionized water, and images were obtained using a Hitachi H-7650 transmission electron microscope.

### BFP isolation.

EPEC strains were harvested from heavily streaked BAP and resuspended in PBS. Samples were vigorously shaken using a Vortex mixer for 1 min and centrifuged at 3,000 × *g* during 30 min. The resulting supernatant solutions were centrifuged an additional 30 min at 17,000 × *g*. BFP were precipitated from this supernatant solution by adding saturated ammonium sulfate to a final concentration of 10%. Precipitated BFP were collected by centrifuging the ammonium sulfate solution at 3,000 × *g* for 30 min. The precipitated BFP subsequently were resuspended in PBS and the protein concentration was quantified. Ten micrograms of total protein was added to each lane of the immunoblot using anti-purified BFP-specific antibody as the primary antibody.

### Autoaggregation assay.

The autoaggregation assay was performed as described by Anantha et al. (20), with some modifications. Bacteria were grown overnight in LB, supplemented with antibiotics as appropriate, at 37°C. The overnight cultures were diluted 1:50 in 4 ml high-glucose DMEM and incubated at 37°C for 4 h at 180 rpm. OD_600_ readings were taken at the end of the incubation period, and the samples then were vigorously shaken using a Vortex mixer for 30 s to disrupt bacterial aggregates. The OD_600_ of the agitated samples was recorded again, and the autoaggregation index at each time point was determined using the formula (AV-OD_600_ − BV-OD_600_)/BV-OD_600_, where BV-OD_600_ and AV-OD_600_ indicate the before and after Vortex-agitated OD_600_ values, respectively.

### Quantitative analysis of surface bundlin expression.

Bacterial strains were grown overnight in LB at 37°C. Overnight cultures then were diluted 1:50 into 5 ml DMEM–F-12 and incubated at 37°C for 3 h with shaking at 180 rpm. An aliquot of cells next was centrifuged for 30 s at 13,000 × *g*, the supernatant solution was discarded, and the cells were resuspended in 100 μl PBS and heat fixed for 30 min using a 65°C heating block. The cells were centrifuged again for 30 s at 13,000 × *g*, resuspended in 200 μl PBS containing a 1:1,000 dilution of rabbit anti-α1-bundlin antibody (32), and incubated on a rotator overnight at 4°C. The samples were centrifuged, and the cell pellets were washed 3 times with PBS and then incubated for 1 h at 25°C with 200 μl PBS containing a 1:1,000 dilution of goat anti-rabbit antibody conjugated to Alexa Fluor 647. The cells were centrifuged again, washed 3 times with PBS, suspended in 200 μl of 1 μM Syto-16, and incubated for 30 min at 25°C. These samples were centrifuged, and the resulting cell pellets were washed once with PBS, suspended in 500 μl of PBS, and analyzed using a BDLSRII flow cytometer (Becton, Dickinson). The data from at least 10^4^ cells were collected using FACSDiva software (Becton, Dickinson).

### Statistics.

Analysis of the eLA and autoaggregation assays was accomplished with GraphPad Prism using one-way analysis of variance (ANOVA) with Tukey's multiple comparison test. Analysis for the flow cytometry assays was performed using a two-tailed *t* test.

## RESULTS

### A mutation in *bfpF* restores piliation in *bfpI*, *bfpJ*, and *bfpK* EPEC mutants.

**FIG 1:**
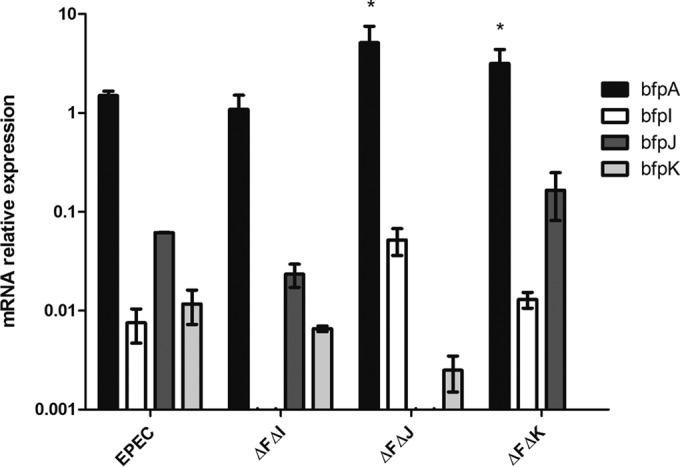
Expression for *bfpA*, *bfpI*, *bfpJ*, and *bfpK* relative to that of the housekeeping gene *rpoA* in wild-type and *bfpF bfpI*, *bfpF bfpJ*, and *bfpF bfpK* EPEC double mutant strains. Relative values are 2^−(Δ*CT*)^. The assay was performed in triplicate three independent times. Error bars represent the standard errors of the means. *, *P* < 0.05 by two-way ANOVA comparing expression to that of WT EPEC.

Investigations into the role of minor pilin-like proteins in the structure and function of different T4aP systems have revealed that although mutant strains possessing single mutations of these genes do not express pili, strains possessing minor pilin-like protein gene mutations in addition to a mutation in the retraction ATPase (PilT) do (22, 33). The results presented in a previous report (19), confirmed in this study, also demonstrated that mutant EPEC strains lacking the ability to express BfpI, BfpJ, or BfpK do not produce BFP. Accordingly, we replaced the *bfpI*, *bfpJ*, and *bfpK* genes with a kanamycin resistance gene in an EPEC *bfpF* deletion mutant to determine if these mutants expressed BFP. The real-time quantitative RT-PCR data presented in Fig. 1 reveal the relative expression of *bfpA*, *bfpI*, *bfpJ*, and *bfpK* mRNA transcripts in the wild-type and mutant strains. These results demonstrate that inserting the Km cassette into each target gene did not affect the mRNA expression from downstream genes, and there was no statistical difference between the expression of the pilin-like genes in the different background strains. As expected from the presence of a Rho-independent transcription terminator sequence downstream of *bfpA*, pilin-like genes are expressed at significantly lower levels relative to that of *bfpA* in wild-type EPEC (34). Unfortunately, attempts to confirm the quantitative RT-PCR results at the translational level using minor pilin-specific antisera were unsuccessful, likely because the concentration of the minor pilins in the whole-cell or semicrude BFP preparations employed in these experiments was below the detection limit of the Western immunoblots.

**FIG 2:**
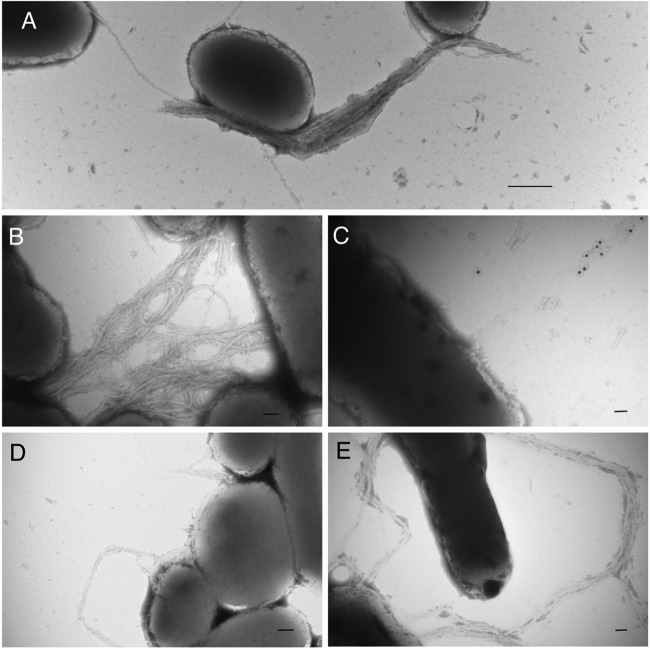
Transmission electron microscope images (TEM) of wild-type EPEC and the double mutant strains grown on BAP overnight. Wild-type EPEC (A) and *bfpF bfpI* (B), *bfpF bfpJ* (C), *bfpf bfpK* (D), and *bfpF* (E) strains are shown. Panel C shows an immunogold-labeled image using the anti-purified BFP antibody. Scale bars represent 500 nm (A) and 100 nm (B, C, D, and E).

**FIG 3:**
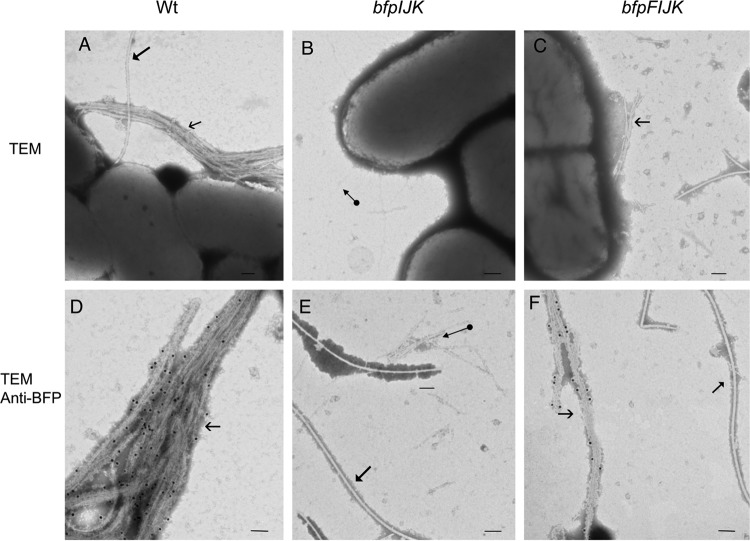
TEM images of WT EPEC, *bfpIJK*, and *bfpFIJK* mutant strains. (D, E, and F) Immunogold labeling (15-nm gold particles) using an anti-BFP polyclonal antibody. The *bfpIJK* triple mutant (B and E) does not produce BFP. Filled arrowheads indicate flagella, open arrowheads indicate BFP, and the circle-tailed arrow indicates different pilus structures (possibly type 1 or common pili) that were not labeled with anti-BFP. Bar size, 100 nm.

TEM analysis (Fig. 2) of the double mutants revealed that the *bfpF* mutant strains lacking the ability to express BfpI, BfpJ, or BfpK were capable of expressing BFP. Remarkably, the BFP expressed by these mutants revealed phenotypic differences from the BFP produced by the single *bfpF* mutant (Fig. 2E) or by the wild-type EPEC strain (Fig. 2A). For example, the pili produced by the *bfpF bfpI* mutant appear to be more tightly entangled than those expressed by the *bfpF* mutant and are longer than the BFP expressed by the wild-type strain (Fig. 2A, B, and E). In contrast, the *bfpF bfpJ* mutant expresses highly fragmented pili, most of which were not attached to bacterial cells and which were detected only by immunogold staining using an anti-purified BFP antibody (Fig. 2C). Finally, the *bfpF bfpK* mutant strain expresses BFP that more closely resembled those produced by the wild-type EPEC strain rather than the *bfpF* mutant (Fig. 2A, D, and E). To determine if the absence of all three pilin-like proteins could affect the expression of BFP, we deleted all three genes from the wild-type and the *bfpF* background strains, thereby creating triple (*bfpIJK*) and quadruple (*bfpFIJK*) mutant strains. TEM analysis revealed that only the quadruple mutant strain produced BFP, but these pili appeared phenotypically similar to the BFP expressed by the *bfpF bfpJ* mutant, appearing shorter and less tangled than those expressed by the *bfpF* mutant (Fig. 3).

**FIG 4:**
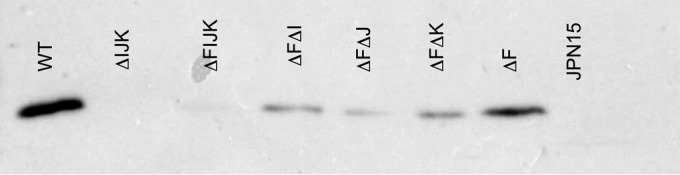
Anti-BfpA Western immunoblot analysis of isolated BFP prepared from WT EPEC mutant strains. JPN15 is an EPEC strain lacking pEAF and represents a negative control on the Western immunoblot.

We also assessed BFP expression by Western immunoblot analysis of bundlin present in isolated pili prepared from all the double as well as the triple and quadruple mutant strains. The results presented in Fig. 4 demonstrate that there was less bundlin in cell-free BFP preparations from all three of the double mutant strains than from their *bfpF* parent strain. As predicted by the TEM images, bundlin was not detected in samples from the triple mutant strain. Although TEM analysis revealed that the quadruple mutant strain expressed BFP, the bundlin signal was barely perceptible on the Western immunoblot presented in Fig. 4. Thus, there is a poor correlation between the TEM of the *bfpF* and *bfpF bfpI* mutants (Fig. 2B and E) and the Western immunoblot results presented in Fig. 4. This apparent discrepancy could be explained by the increased physical entanglement observed in some mutants, which may have caused a few of these pili to remain in the supernatant solution during the purification process, which relied on centrifugation to separate sheared BFP from the bacterial cells. Moreover, in the absence of all three minor pilins in the quadruple mutant, pseudopilins of the type 2 secretion system may prime for BFP assembly (35). However, the type 2 secretion system pseudopilins may not perfectly substitute for the priming function of BfpI, BfpJ, or BfpK, thereby leading to the reduced bundlin signal in the quadruple mutant.

**FIG 5:**
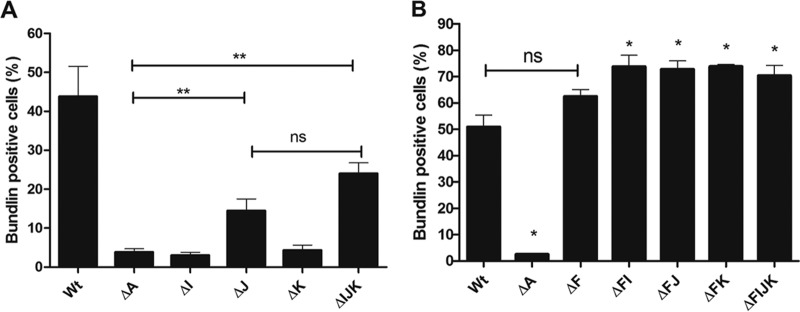
Flow cytometry quantitative analysis of surface-exposed bundlin. Surface-exposed bundlin was detected by flow cytometry and expressed as the percentage of Alexa Fluor 647 fluorescence-positive cells also displaying Syto-16 fluorescence above background levels. (A) Surface expression of bundlin in WT and *bfpA*, *bfpI*, *bfpJ*, *bfpK*, and *bfpIJK* mutant strains. (B) Surface expression of bundlin in strains shown in panel A with a mutation in *bfpF*. Error bars indicate standard errors of the means from three independent experiments (**, *P* < 0.03 relative to the *bfpA* mutant; *, *P* < 0.03 relative to the wild-type strain; ns, not significant).

Using flow cytometry, we next investigated the percentage of pilin-like mutant cells that expressed immune-reactive bundlin on their surfaces. This assay does not distinguish between unassembled bundlin and bundlin assembled into BFP. The data presented in Fig. 5 reveal that a greater percentage of the double mutant strains expressed surface bundlin than the wild-type strain. In addition, the quadruple mutant strain was capable of expressing bundlin on the surface of the bacteria (Fig. 5). These results also revealed that although we did not detect intact BFP on the single *bfpJ* and the triple *bfpIJK* mutant strains by TEM analysis, a significant percentage of these cells did express surface-exposed bundlin.

### *bfpF bfpI* and *bfpF bfpJ* mutants do not adhere to HEp-2 cells.

**FIG 6:**
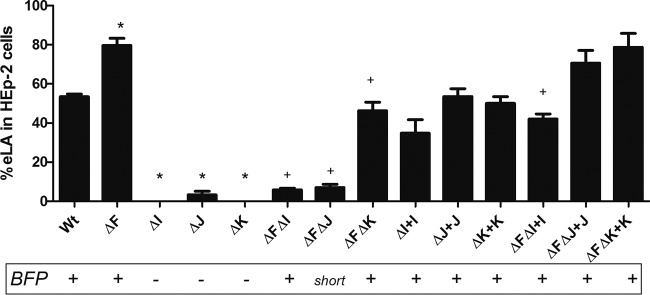
Early localized adherence (eLA) assays performed with wild-type and T4bP mutant EPEC. WT EPEC, *bfpF* (ΔF), *bfpI* (ΔI), *bfpJ* (ΔJ), *bfpK* (ΔK), *bfpF bfpI* (ΔFΔI), *bfpF BfpJ* (ΔFΔJ), *bfpF bfpK* (ΔFΔK), *bfpI*+pT3BfpI (ΔI+I), *bfpJ*+pT3BfpJ (ΔJ+J), *bfpK*+pT3BfpK (ΔK+K), *bfpF bfpI*+pT3BfpI (ΔFΔI+I), *bfpF bfpJ*+pT3BfpJ (ΔFΔJ+J), and *bfpF bfpK*+pT3BfpK (ΔFΔK+K) strains are shown. The presence or absence of BFP, as revealed by TEM analysis, is indicated below the graph. The assays were performed by counting HEp-2 cells with EPEC microcolonies (at least 4 or more aggregated bacteria) in 10 fields in triplicate three separate times, and the error bars represent the standard deviations from the means. An asterisk indicates a significant difference (*P* < 0.001 by one-way ANOVA) between the single mutant strains and the WT, and a plus sign indicates a significant difference between the double mutant and the *bfpF* mutant strains.

It has been shown that only EPEC strains harboring the EAF plasmid express the LA phenotype, which was attributed to the expression of BFP. Therefore, we investigated whether the BFP produced by the pilin-like double mutants were functional in adherence to HEp-2 cells. Despite its piliated phenotype, the *bfpF bfpI* mutant displayed low eLA (see Materials and Methods) to HEp-2 cells (5%) relative to that for the *bfpF* mutant strain (80%) and the wild-type strain (52%) (Fig. 6). The *bfpF bfpJ* mutant also displayed significantly reduced eLA (7%). In contrast, the *bfpF bfpK* mutant adhered to HEp-2 cells better than either of the *bfpF bfpI* or *bfpF bfpJ* strains but less so than the *bfpF* deletion mutant in which it was created. Complementing the mutants with the *bfpI* or *bfpJ* gene (in *trans*) fully (*bfpF bfpJ*) or almost completely (*bfpF bfpI*; from 5% to 39%) restored the ability of the organisms to adhere to HEp-2 cells relative to that of the *bfpF* parent strain.

**FIG 7:**
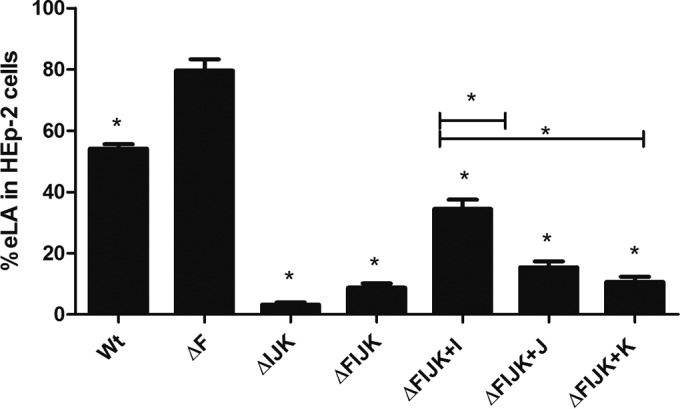
HEp-2 cell eLA of the WT, the quadruple mutant EPEC, and respective complemented strains. The assay was performed three times, each time in triplicate, allowing the strains to interact with the HEp-2 cells for 45 min. Error bars indicate standard errors of the means. *, *P* < 0.001 by one-way ANOVA relative to the EPEC ΔF strain and between the complemented strains.

To determine which of the three pilin-like proteins were critical to the EPEC eLA phenotype, we assessed the ability of the quadruple mutant and the corresponding complemented strains to adhere to HEp-2 cells. eLA of the *bfpFIJK* mutant strain was significantly diminished to 9% (Fig. 7), whereas it was 80% eLA for the *bfpF* mutant. Complementing the quadruple mutant strain using the pMPM-T3 plasmid containing either the *bfpI*, *bfpJ*, or *bfpK* gene individually revealed that *bfpI* significantly restored the ability of the *bfpFIJK* mutant to adhere to HEp-2 cells, increasing its eLA from 9% to 34% (Fig. 7). Although eLA appeared to increase when *bfpJ* or *bfpK* was reintroduced into the quadruple mutant, this increase was not significantly different relative to that displayed by the uncomplemented quadruple mutant strain (15% and 10%, respectively) but was significantly different from that of the *bfpI*-complemented strain.

### Absence of BfpI and BfpJ affects autoaggregation in the EPEC *bfpF* mutant.

**FIG 8:**
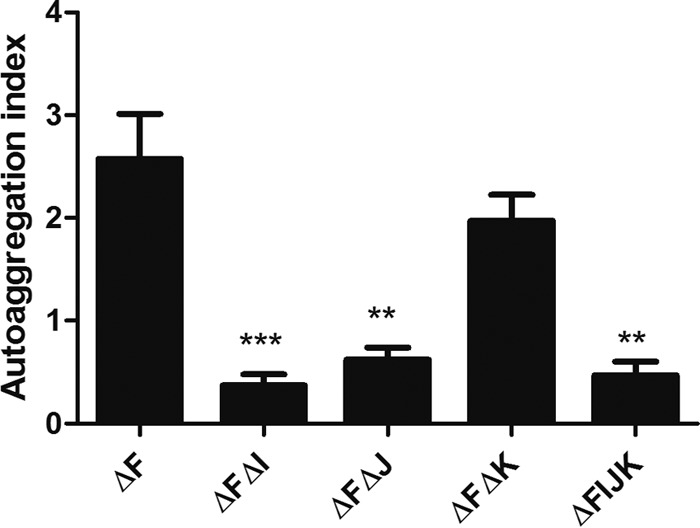
Autoaggregation index of EPEC mutants. Autoaggregation index of *bfpF* (ΔF), *bfpF bfpI* (ΔFΔI), *bfpF bfpJ* (ΔFΔJ), *bfpF bfpK* (ΔFΔK), and *bfpFIJK* (ΔFIJK) strains is shown. The assay was performed in triplicate. Error bars indicate standard errors of the means (*P* < 0.001 [**] and *P* < 0.0001 [***] for significant differences relative to the ΔF strain by one-way ANOVA with Tukey's multiple-comparison test).

It was previously reported (20, 22) that T4aP and T4bP retraction ATPase mutants of both EPEC (*bfpF*) and N. meningitidis (*pilT*) display a greater autoaggregation phenotype than wild-type organisms. Consequently, we determined the autoaggregation phenotype (Fig. 8) of the double mutant in addition to the quadruple mutant strains and compared it to that of the *bfpF* parent mutant. The absence of BfpI from the *bfpF bfpI* mutant resulted in a significant (*P* < 0.0001 by one-way ANOVA) reduction in its autoaggregation index relative to that of the *bfpF* parent strain. In contrast, the *bfpF bfpJ* double mutant and the quadruple mutant strains displayed significantly (*P* < 0.001 by one-way ANOVA) less of a reduction in their autoaggregation indices than the *bfpF* strain (0.6 and 0.4 versus 2.9, respectively).

### BfpI, BfpJ, and BfpK are incorporated into the BFP filament.

**FIG 9:**
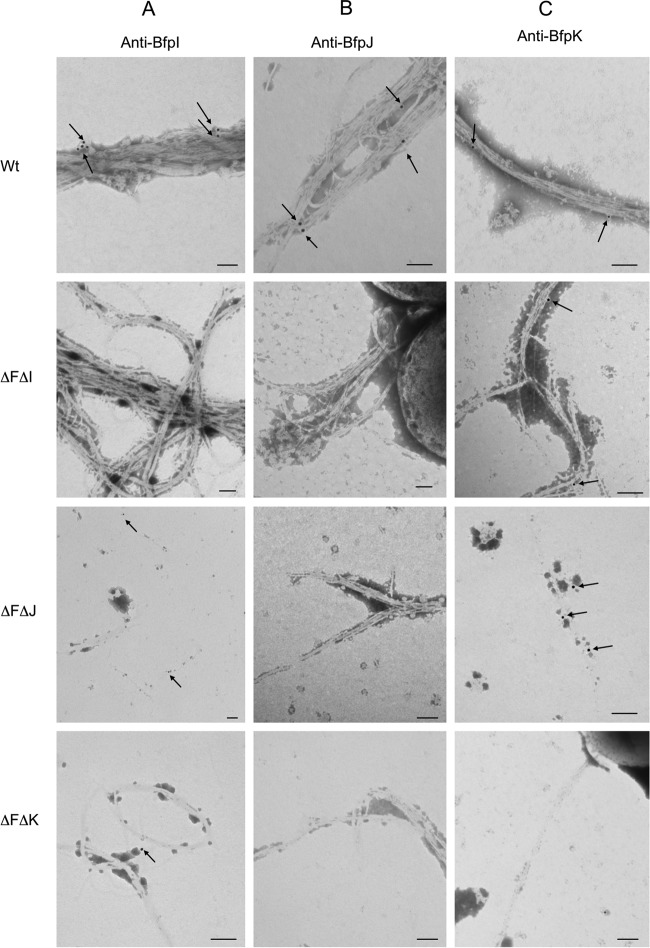
Immunogold (arrows) labeling of BfpI, BfpJ, and BfpK in BFP from wild-type EPEC and minor pilins mutants. (A) Anti-BfpI label; (B) anti-BfpJ label; (C) anti-BfpK label. Bars, 100 nm; immunogold particle size, 15 nm.

Since BfpI, BfpJ, and BfpK share structural similarities with bundlin (1) (see Fig. S1 and S2 in the supplemental material), we used the TEM immunogold labeling technique to investigate whether these subunits are coassembled along with bundlin into the BFP filaments. As demonstrated by the images presented in Fig. 9, we detected immunogold labeling of BfpI, BfpJ, and BfpK subunits in the wild-type BFP but not in their respective *bfpF bfpI*, *bfpF bfpJ*, or *bfpF bfpK* double mutant BFP filaments. Moreover, TEM analysis (Fig. 9) revealed that although BfpI and BfpK pilin-like proteins were detected in BFP expressed in the *bfpF bfpJ* mutant strain, we could not detect BfpJ in either the *bfpF bfpI* or *bfpF bfpK* mutant strain.

## DISCUSSION

The structural and functional roles of minor pilins have been studied mostly in T4aP systems and just recently in the T4bP expressed by enterotoxigenic E. coli (22, 36–38). The BFP of enteropathogenic Escherichia coli represent a model of T4bP, and here we report the findings of the role of the minor pilin-like proteins in these pili. In the present study, we confirmed that whereas single mutants in *bfpI*, *bfpJ*, and *bfpK* pilin-like genes do not produce BFP, Western immunoblot and TEM analyses revealed that mutants created in an EPEC *bfpF* background strain do. Moreover, immunogold labeling revealed that all three of the pilin-like subunits (BfpI, BfpJ, and BfpK) are incorporated along with bundlin (BfpA) into the wild-type BFP filament and are important for BFP assembly and function (Fig. 4 and 9). It would seem appropriate, therefore, to refer to these subunits as minor pilins as reported in the T4aP expressed by N. meningitidis and P. aeruginosa (21, 39).

TEM analysis of immunogold-labeled preparations also revealed that the BfpI and BfpK minor pilins were incorporated into BFP produced in the *bfpF bfpJ* mutant strain, but we could not detect BfpJ in BFP expressed by either the *bfpF bfpI* or *bfpF bfpK* mutant strains (Fig. 9). Given that BfpJ was readily detected by immunogold labeling in BFP expressed by wild-type EPEC (Fig. 9), the greatly reduced or absent BfpJ immunogold labeling in the BFP expressed by the *bfpF bfpI* and *bfpF bfpK* mutant EPEC strains (Fig. 9) is most consistent with the conclusion that, in the absence of either BfpI or BfpK, BfpJ is not incorporated into BFP. It is also consistent with the observation (Fig. 7) that the presence of BfpJ alone had little positive influence on the host cell adhesion properties of BFP.

TEM analysis also revealed that, relative to BFP expressed by the wild-type or *bfpF* mutant strains, the *bfpF bfpJ* double mutant strain produced what appeared to be fragmented BFP, which were difficult to detect on the EM grids unless we employed the immunogold labeling technique. Moreover, many of these BFP fragments did not appear to be attached to the bacterial cells. Despite this, the Western immunoblot results presented in Fig. 4 demonstrated that the *bfpF bfpJ* double mutant expressed what appeared to be the same amount of BFP as the *bfpF bfpI* and *bfpF bfpK* double mutant strains. These observations suggest that BFP lacking BfpJ subunits is more prone to breakage than BFP expressed by the *bfpF* or wild-type EPEC strains. Therefore, BfpJ, perhaps in association with BfpI or BfpK, may contribute to the structural integrity of wild-type BFP, making them less prone to breakage. Nonetheless, despite its tendency to produce fragmented BFP, the *bfpF bfpJ* double mutant strain demonstrated low, but still detectable, levels of eLA to HEp-2 cells in addition to its autoaggregation phenotype (Fig. 6 and 8), suggesting that BFP lacking BfpJ that remained attached to the bacterial cell surface partially maintained their ability to promote adhesion.

Previously, we reported that bundlin displayed lectin-like activity, demonstrating specificity for *N*-acetyl-d-lactosamine glycan receptors on host cells (30, 40). Although TEM analysis revealed that the *bfpF bfpI* double mutant appeared to express BFP similar to that of the *bfpF* parental mutant, this strain displayed a significantly reduced ability to autoaggregate (Fig. 8) or adhere to HEp-2 cells (Fig. 6). These observations are reminiscent of those reported for the minor pilin PilX. The N. meningitidis *pilX* mutant expresses T4aP but is completely unable to adhere to or to form microcolonies on host cells (39, 41). Our results suggest that the absence of BfpI from BFP filaments either compromises the ability of bundlin to ligate its receptors on HEp-2 cells or that BfpI also serves a direct role, perhaps as a coadhesin, in the host cell recognition process. However, we cannot exclude the possibility that BfpJ also contributes to the BFP host cell adhesive function, since BfpJ also is lacking in the BFP expressed by the *bfpF bfpI* mutant strain (Fig. 9). More detailed structural analysis of BfpI, BfpJ, and the BFP produced by the *bfpF bfpI* double mutant is required to complete our understanding of how the conformation or assembly of BFP is affected when BfpI and BfpJ are absent.

With respect to BfpK, it would appear that, in the absence of retraction, this subunit is dispensable without compromising either the BFP structure, at least at the level of resolution of TEM, or eLA function (Fig. 6). However, the TEM observations revealed that, unlike the *bfpF* parent strain, the *bfpF bfpK* mutant appears to express BFP that more closely resemble in number those produced by the wild-type organisms. This suggests that BfpK, possibly in association with BfpJ (Fig. 9), is involved in regulating the BFP extension/retraction processes. In addition, TEM analysis revealed that the quadruple *bfpFIJK* mutant strain expressed BFP which appeared to be similar to those expressed by the *bfpF bfpJ* double mutant. It has been reported that pseudopilins of the type 2 secretion system may substitute for the minor pilins in priming T4P assembly in Pseudomonas aeruginosa (36). Further studies will be required to determine whether, in the absence of all three (BfpI, BfpJ, and BfpK) minor pilins, pseudopilins play a similar role in EPEC BFP expression (35, 42).

As anticipated, flow cytometry analysis, using a bundlin-specific polyclonal antibody, revealed a low percentage of bundlin-positive cells in the *bfpA* mutant population, representing background levels (Fig. 5A). Analysis of the *bfpI* and *bfpK* single mutant strains produced an equivalent result. In contrast, the percentage of bundlin-positive cells was significantly greater in the *bfpJ* and *bfpIJK* mutant strains than in the *bfpA* mutant. Despite this, TEM analysis did not reveal BFP in either of these mutants. These observations imply that although the *bfpJ* single and *bfpIJK* triple mutant strains appear not to express BFP that could be detected by TEM, a significant percentage of these cells have surface bundlin. We cannot exclude the presence of very short truncated pilus structures, perhaps associated with the BFP outer membrane assembly apparatus, or the possibility of unassembled bundlin on the outer membranes of the organisms. This suggests that BfpJ serves a role in regulating BFP assembly in addition to stabilizing the pilus filaments, as suggested by the image presented in Fig. 2C.

The flow cytometry technique also revealed that a significantly higher percentage of cells from all three double mutant strains and from the quadruple mutant expressed unassembled surface bundlin relative to percentages for the wild-type strain. Despite these results, TEM analysis did not reveal any apparent increase but rather the opposite in BFP expression by either the *bfpF bfpJ*, *bfpF bfpK*, or *bfpFIJK* mutant strain. TEM, however, would not have the same capability as the flow cytometry technique to detect immune-reactive short truncated BFP or unassembled bundlin on the surface of the bacteria. These observations do suggest, though, that apparent phenotypic differences in the pili expressed by the double mutant strains are due not to defects in the processing and transport of bundlin to the outer membrane BFP assembly apparatus but rather to pilus polymerization and stability. Furthermore, these results reveal a fundamental difference between the *bfpJ* and the *bfpI* and *bfpK* single mutants, since we could detect bundlin on the surface of the *bfpJ* single mutant strain but not in the *bfpI* or *bfpK* single mutants, while TEM analysis did not reveal assembled BFP in any of these single mutant strains. The ability of double retraction-deficient, minor pilin mutant strains to make T4P while single minor pilin strains cannot has been interpreted as evidence that minor pilins facilitate pilus extension, impede retraction, or both. Whatever the function with regard to the relative efficiency of pilus extension and retraction, it appears that BfpJ plays less of a role than the other pilin proteins, since bundlin monomers still can reach the surface in the *bfpJ* mutant but not in the *bfpI* or *bfpK* mutants. Moreover, analysis of the triple *bfpIJK* mutant indicates that the net effect of losing all three minor pilins still permits some bundlin to reach the bacterial cell surface.

Although it is tempting to use our immunogold labeling results to estimate the number of BfpI, BfpJ, and BfpK subunits incorporated into the BFP filaments relative to bundlin, this would require using antibodies with avidities similar to those of their respective antigens. However, enzyme-linked immunosorbent assay (ELISA) results (data not shown) indicate that the BfpI-, BfpJ-, and BfpK-specific antisera used in the immunogold labeling experiments do not fulfill this requirement. Moreover, because they form dense rope-like structures, it is also extremely challenging to determine the relative frequency or localization of BfpI, BfpJ, or BfpK incorporation into individual BFP filaments.

In sum, here we present evidence that the minor pilins BfpI, BfpJ, and BfpK are incorporated into BFP expressed by EPEC. BfpI might have a direct role as a coadhesin or indirectly participate in maintaining the BFP host cell recognition properties. The results also suggest that BfpJ is required to maintain BFP flexibility or tensile strength, and that BfpK regulates either the extension or the retraction phase of BFP expression.

## Supplementary Material

Supplemental material
