# Supplementary material for: BfpI, BfpJ, and BfpK Minor Pilins Are Important for the Function and Biogenesis of Bundle-Forming Pili Expressed by Enteropathogenic Escherichia coli
Source: J Bacteriol. 2016 Feb 12;198(5):846–56. doi: 10.1128/JB.00818-15 (PMC4810605; doi:10.1128/JB.00818-15)
Supplement: Supplemental material [file supp_198_5_846__index.html]

Supplemental material 

# BfpI, BfpJ, and BfpK Minor Pilins Are Important for the Function and Biogenesis of Bundle-Forming Pili Expressed by Enteropathogenic Escherichia coli

## Supplemental material

- Supplemental file 1 -

  Fig. S1, secondary structure prediction

  Fig. S2, sequence alignment

  PDF, 606K
