# Supplementary material for: BfpI, BfpJ, and BfpK Minor Pilins Are Important for the Function and Biogenesis of Bundle-Forming Pili Expressed by Enteropathogenic Escherichia coli
Source: J Bacteriol. 2016 Feb 12;198(5):846–56. doi: 10.1128/JB.00818-15 (PMC4810605; doi:10.1128/JB.00818-15)
Supplement: Supplemental material [file JB.00818-15_zjb999093950so1.pdf]

Supplementary material

BfpI secondary Structure

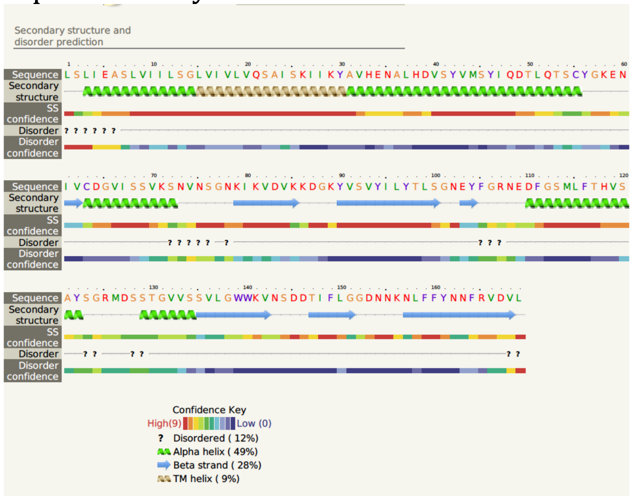

BfpJ secondary structure

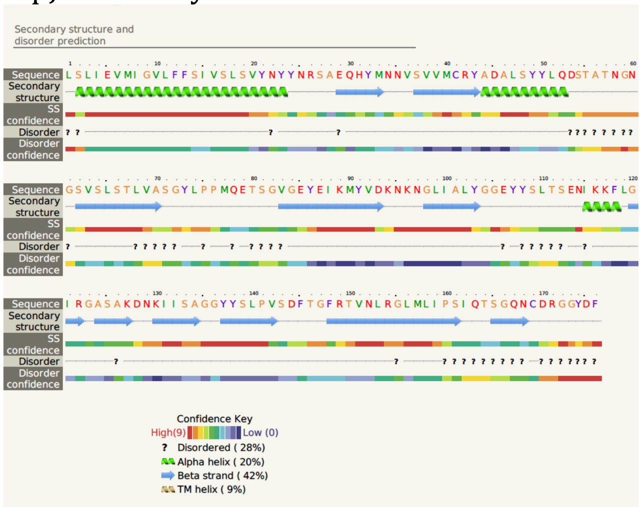

BfpK secondary structure

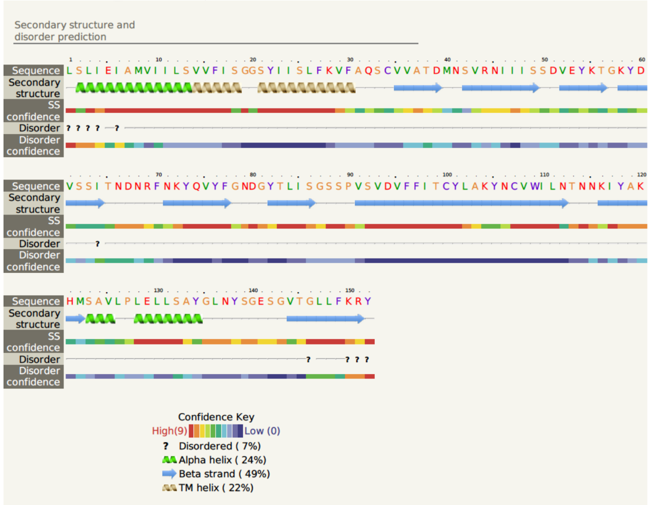

Figure S1. **Secondary structure prediction of BfpI, BfpJ and BfpK.** Prediction of secondary structure of mature BfpI, BfpJ and BfpK minor pilins. Modeling was done using Phyre2, showing the presence of alpha helix in the hydrophobic N-terminal.

```
BfpI            -MFILRNKRKESGLSLIEASLVIILSGLVIVLVQSAISKIIKYAVHENALHDVS----YV 55
BfpK            ---MIFEGRQQKGLSLIEIAMVIILSVVFISGGSYIISLFKVFAQSCVVATDMN----SV 53
BfpA            MVSKIMNKKYEKGLSLIESAMVLALAATVTAGVMFYYQSASDSNKSQNAISEVM----SA 56
BfpJ            ------MIRKESGLSLIEVMIGVLFFSIVSLSVYNYYNRSAEQHYMNNVSVVMCRYADAL 54
                        : :.******  : : :   .        .          .   :

BfpI            MSYIQDTLQTSCYGKENIVCDGVISSVKSNVNSGNKIKVDVKKDGKYVSVYILYTLSGNE 115
BfpK            RNIIISSDVEYKTGKYDVSS-------ITNDNRFNKYQVYFGNDG-YTLISGSSPVSVDV 105
BfpA            TSAINGLYIGQTSYSGLDSTILLNTSAIPDNYKDTTNKKITNPFGGELNVGPANNNTAFG 116
BfpJ            SYYLQDSTATNGNGSVSLSTLVASGYLPPMQETSGVGEYEIKMYVDKNKNGLIALYGGEY 114
                   : .        .             .        :

BfpI            YFGRNEDFGSMLFTHVSAYSGRMDSS--------TGVVSSVLGWWKVNSDDTIFLGGDNN 167
BfpK            FFIT---CYLAKYNCVWILNTNNKIY--------AKHMSAVLPLELLSAYGLNYSGESGV 154
BfpA            YYLTLTRLDKAACVSLATLNLGTSAKGYGVNISGENNITSFGNSADQAAKSTAITPAEAA 176
BfpJ            YSLTSENIKKFLGIRGASAKDNKIISAGG---YYSLPVSDFTGFRTVNLRGLMLIPSIQT 171
                :                  .                 :: .         .

BfpI            KNLFFYNNFRVDVL--- 181
BfpK            TGLLFKRY--------- 162
BfpA            TACKNTDSTNKVTYFMK 193
BfpJ            SGQNCDRGGYDF----- 183
                .
```

Figure S2. Alignment of BfpA, BfpI, BfpJ and BfpK sequences.

The signal peptide is show in Red letters, the Gly residue where BfpP cuts the leader peptide is shown in red. All three mature proteins start with Leu and all three have the Glu conserved at position 5. In green the conserved **C** residues in the C-terminal regions are shown. In yellow is where the truncated protein was cloned for purification in the present study.
